# Basal Autophagy Is Necessary for A Pharmacologic PPARα Transactivation

**DOI:** 10.3390/cells11040754

**Published:** 2022-02-21

**Authors:** Eun Young Kim, Jae Man Lee

**Affiliations:** 1 Department of Biochemistry and Cell Biology, Cell and Matrix Research Institute, School of Medicine, Kyungpook National University, Daegu 41944, Korea; key11@knu.ac.kr; 2 BK21 FOUR KNU Biomedical Convergence Program, Department of Biomedical Science, The Graduate School, Kyungpook National University, Daegu 41944, Korea

**Keywords:** basal autophagy, PPARα, ATG7, NRF2, KEAP1, transactivation, gene expression

## Abstract

Autophagy is a conserved cellular process of catabolism leading to nutrient recycling upon starvation and maintaining tissue and energy homeostasis. Tissue-specific loss of core-autophagy-related genes often triggers diverse diseases, including cancer, neurodegeneration, inflammatory disease, metabolic disorder, and muscle disease. The nutrient-sensing nuclear receptors peroxisome proliferator-activated receptor α (PPARα) plays a key role in fasting-associated metabolisms such as autophagy, fatty acid oxidation, and ketogenesis. Here we show that autophagy defects impede the transactivation of PPARα. Liver-specific ablation of the *Atg7* gene in mice showed reduced expression levels of PPARα target genes in response to its synthetic agonist ligands. Since NRF2, an antioxidant transcription factor, is activated in autophagy-deficient mice due to p62/SQSTM1 accumulation and its subsequent interaction with KEAP1, an E3 ubiquitin ligase. We hypothesize that the nuclear accumulation of NRF2 by autophagy defects blunts the transactivation of PPARα. Consistent with this idea, we find that NRF2 activation is sufficient to inhibit the pharmacologic transactivation of PPARα, which is dependent on the *Nrf2* gene. These results reveal an unrecognized requirement of basal autophagy for the transactivation of PPARα by preventing NRF2 from a nuclear translocation and suggest a clinical significance of basal autophagy to expect a pharmacologic efficacy of synthetic PPARα ligands.

## 1. Introduction

Autophagy is an evolutionarily conserved catabolic and adaptive process induced by diverse conditions, including the deprivation of nutrient or growth factors, pathogen infection, hypoxia, and even exercise [1,2,3,4,5,6,7]. This leads to nutrient recycling, prevents cellular damage, and maintains tissues homeostasis in response to energy shortage and constant external and internal harmful insults. Therefore, autophagy has inevitable cytoprotective roles and needs to be precisely and tightly controlled to properly respond to various stimuli. It has been shown that autophagy dysfunctions are intimately associated with a wide range of human pathogenesis, including neurodegeneration, inflammatory and metabolic diseases, and cancers [3,8,9]. Recent studies reveal that autophagy used to be considered a nonselective process is able to selectively eliminate unwanted, potentially cytotoxic molecules, such as damaged organelles or aggregated proteins, serving as a primary cytoprotective function [10,11]. This leads to intensive research to modulate autophagy activity due to its therapeutic potential in many human diseases [12].

Macroautophagy is the major type of autophagy and has been most intensively studied. However, it is important to note that there are two other types of autophagy: microautophagy and chaperone-mediated autophagy. The initial genetic screen in yeasts identified many core autophagy-related genes. Subsequent studies have found that they are highly conserved in mammals, including mice and humans. For example, Atg1 and Atg6 in yeast are homologs of mammalian ULK1 and Beclin1, respectively [13].

In particular, tissue-specific *Atg7* ablation using a Cre-loxp system in mice has revealed that core autophagy-related genes encoding macroautophagy machinery proteins are essential for maintaining almost every tissue homeostasis [14,15,16,17,18,19,20]. It has also been shown that liver-specific *Atg7* (*Atg7^LKO^*) knockout mice showed hepatomegaly phenotypes and marked accumulations of p62, an autophagy adaptor protein [17,21]. Increased protein levels of p62 are capable of interacting with KEAP1, an E3 ubiquitin ligase, which liberates NRF2 from the ubiquitin-mediated proteasomal degradation system. As a potent antioxidant transcription factor, the nuclear NRF2 binds to the antioxidant response element (ARE) of many cytoprotective genes such as *Nqo1*, *Hmox1*, *Gsta1*, and so forth and upregulates their expressions to remove reactive oxygen species [22,23].

Among 48 members of nuclear receptors in the human genome, PPARα is the most notable nuclear receptor activated during fasting and regulates the expression of genes involved in fasting-mediated metabolism, including fatty acid oxidation and ketogenesis [24,25,26,27]. Consistent with its essential roles during fasting, Lee et al. showed that PPARα is necessary for fasting-induced autophagy and that pharmacological activation of PPARα is sufficient for induction of autophagy even in fed state [28,29]. Particularly, PPARα plays a key role in the induction of lipophagy, a selective autophagy for lipid droplets. This may provide peroxisomes and mitochondria with free fatty acids for β-oxidation and ketogenesis [28,29].

In this study, we want to define whether autophagy activity affects a pharmacological activation of nuclear receptor PPARα. A previous study has already suggested that both *Atg7* knockdown in AML12 cells and *Atg7^LKO^* mice showed reduced concentrations of ketone body in response to synthetic PPARα agonists [28]. This result allows us to further investigate whether basal autophagy is essential for the pharmacologic activation of PPARα. We found that autophagy inhibition in vivo and in cultured cells blunts pharmacological induction of many PPARα target genes. Furthermore, as it has been reported that there are crosstalks between NRF2 and PPARγ, the closest homolog of PPARα in various tissues [30,31,32,33,34], NRF2 activation is often observed in conditions of autophagic defects might interfere with the pharmacological activation of PPARα in the nucleus.

## 2. Materials and Methods

### 2.1. Chemicals and Reagents

Wild-type C57BL/6J mice were purchased from Japan SLC, Inc. (Hamamatsu, Shizuoka, Japan) (C57BL/6JJmsSlc); *Atg7^F/F^* (RBRC02759) [17] mice were purchased from RIKEN BioResource Research Center (Tsukuba, Ibaraki, Japan); *Alb-Cre* mice (strain #003574) [35] and *Nrf2^F/F^* mice (strain #025433) [29,36] were purchased from Jackson laboratory (Bar Harbor, ME, USA); *Keap1^F/F^* mice [37] were obtained from Masayuki Yamamoto laboratory. AML12 cells were purchased from ATCC (Manassas, VA, USA) (CRL-2254); GW7647 (Cat. #10008613), Wy-14,643 (Cat. #70703), and L-sulforaphane (SFN, Cat. #14797) from Cayman; dimethylfumarate (DMF, Cat. #242926), butylated hydroxyanisole (BHA, Cat. #B1253-500G), dexamethasone (Cat. #D4902), polyethylene glycol 400 (PEG 400, Cat. #P3265-1KG), 3-methyladenine (Cat. #M9281) and Tween 80 (Cat. #P1754-500ML) from Sigma-Aldrich (St. Louis, MO, USA); Bafilomycin A1 (BafA1, Cat. #BML-CM110) from Enzo Life Science (Farmingdale, NY, USA); HyClone DMEM/F12 (Cat. #SH30023.01) and fetal bovine serum (FBS, Cat. #SH20084.03) from HyClone (Logan, UT, USA); Trizol Reagent (Cat. #15596018) from Invitrogen (Waltham, MA, USA); RbTaq^TM^ qPCR 2X PreMIX (SYBR Green with high ROX, Cat. #RT531M) from Enzynomics (Daejeon, Korea); PrimeScript^TM^ 1st strand cDNA Synthesis kit (Cat. #6110A) from TaKaRa Bio Inc. (Kusatsu, Shiga, Japan); cOmplete^TM^, Mini, EDTA-free Protease Inhibitor Cocktail (Cat. #11836170001), PhosSTOP^TM^ (Cat. #04906837001), and Insulin-Transferrin-Sodium Selenite Supplement (ITS, Cat. #11074547001) from Roche (Basel, Switzerland); penicillin-streptomycin (Cat. #15140122), Pierce^TM^ BCA protein assay kit (Cat. #23225) and Pierce ECL Western Blotting Substrate (Cat. #32106) from Thermo Scientific (Waltham, MA, USA); BSA (Cat. #0332-100G) from VWR (Radnor, PA, USA); Immun-Blot PVDF Membrane (Cat. #1620177) from Bio-Rad Laboratories (Hercules, CA, USA); Rabbit monoclonal anti-β-actin (13E5) antibody (Cat. #5125, 1:3000 dilution), Rabbit monoclonal anti-Atg7 (D12B11) antibody (Cat. #8558S, 1:1000 dilution), Rabbit SQSTM1/p62 antibody (Cat. #5114S, 1:1000 dilution) and anti-rabbit IgG HRP-linked antibody (Cat. #7074S, 1:3000 dilution) from Cell Signaling Technology (Danvers, MA, USA); Dimethyl sulfoxide (DMSO, Cat. #sc-358801) from Santa Cruz Biotechnology, Inc. (Dallas, TX, USA); *Atg5^−/−^* (RCB 2711) and *Atg7^−/−^* (RCB 3707) MEFs from Riken BRC. Information for other reagents not shown here is described in the relevant methods and references.

### 2.2. Animal Studies

**Figure 1:**
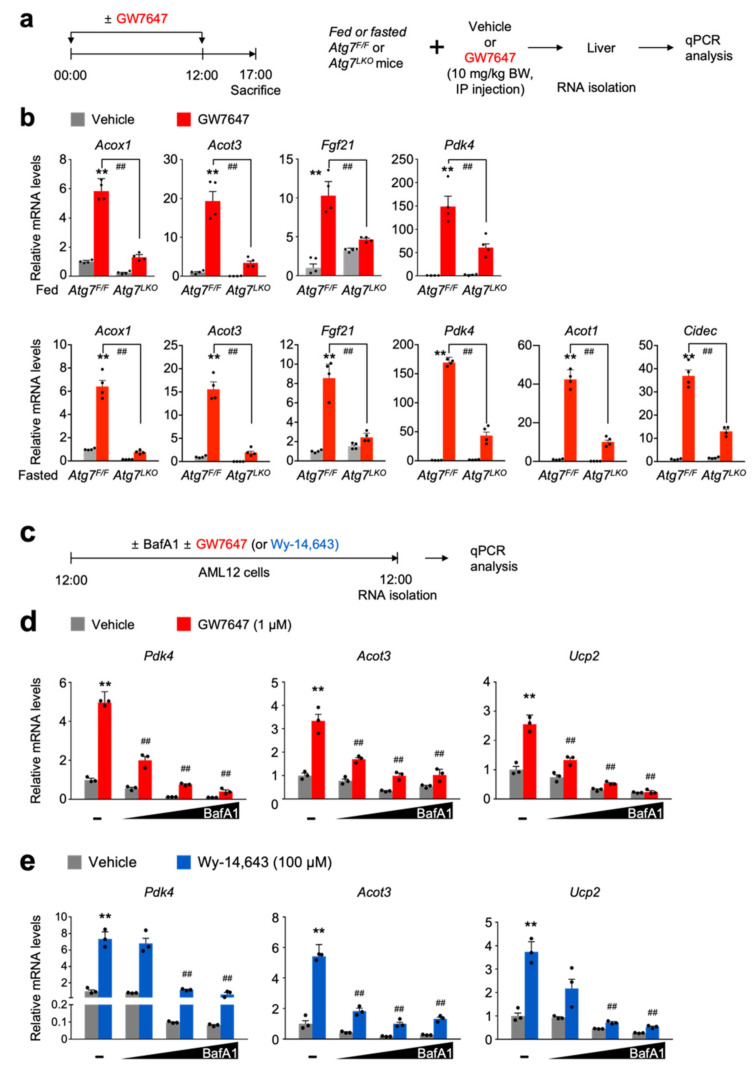
Autophagy inhibition impairs pharmacologic PPAR𝛼 transactivation. (**a**) A schematic diagram of an experimental procedure in mice. Fed or 24 h fasted *Atg7^F/F^*, and *Atg7^LKO^* mice were intraperitoneally injected with vehicle (0.1% DMSO in 4:1 ratio of PEG 400 and Tween 80) or GW7647, a synthetic PPAR𝛼 agonist (10 mg/kg BW) twice a day. A total of 5 h after last treatment, all mice were killed to collect livers, of which total RNAs were isolated for qPCR analysis. (**b**) Hepatic expression levels of PPAR𝛼 target genes *Acox1*, *Acot3*, *Fgf21*, *Pdk4*, *Acot1,* and *Cidec* were determined in *Atg7^F/F^* and *Atg7^LKO^* mice shown in panel (**a**) by qPCR analysis. *n* = 4 per group, ** p < 0.01 vs. *Atg7^F/F^* mice treated with vehicle. *^##^ p* < 0.01. Data represent mean ± s.e.m. and are plotted as fold change. Each dot indicates an individual mouse. Statistics by two-tailed *t*-test. (**c**) A schematic diagram of an experimental procedure in AML12 cells, a mouse hepatocyte-derived cell line. AML12 cells were treated with bafilomycin A1 (BafA1: 0.1, 0.5, or 1 μM), a known autophagy inhibitor in the absence or presence of synthetic PPAR𝛼 agonists (1 μM of GW764 or 100 μM of Wy-14,643) for 24 h. Vehicle is 0.1% DMSO. Total RNAs from these cells were prepared to perform qPCR analysis. (**d**,**e**) Expression levels of PPAR𝛼 target genes *Pdk4*, *Acot3*, and *Ucp2,* were determined in AML12 cells shown in panel (**c**) by qPCR analysis. *n* = 3 per group, ** p < 0.01 vs. AML12 cells treated with vehicle. *^##^ p* < 0.01 vs. AML12 cells treated with GW7647 or Wy-14,643. Data represent mean ± s.e.m. and are plotted as fold change. Each dot indicates an individual sample. Statistics by a two-tailed, unpaired Student *t*-test. BW, body weight.

All animal studies and procedures were approved by the Institutional Animal Care and Use Committee of the Kyungpook National University (KNU-2020-036). Wild-type mice were C57BL/6J mice. Male *Alb-Cre/+* mice were first bred with either female *Atg7^F/F^*, *Keap1^F/F^*, or *Nrf2^F/F^* mice to generate male *Alb-Cre*; *Atg7^F/+^*, *Alb-Cre*; *Keap1^F/+^*, or *Alb-Cre*; *Nrf2^F/+^* mice. These mice were then further bred with female *Atg7^F/F^*, *Keap1^F/F^*, or *Nrf2^F/F^* mice to generate *Alb-Cre*; *Atg7^F/F^* (*Atg7^LKO^*), *Alb-Cre*; *Keap1^F/F^* (*Keap1^LKO^*), or *Alb-Cre*; *Nrf2^F/F^* mice (*Nrf2^LKO^*) that showed a hepatocyte-specific ablation of either *Atg7*, *Keap1*, or *Nrf2* gene, respectively. All experiments were performed in ad libitum fed male mice unless otherwise indicated. To activate PPARα in the liver, 8–9 week-old male wild-type C57BL/6J, *Atg7^F/F^*, *Atg7^LKO^*, *Alb-Cre/+*, *Keap1^LKO^*, *Nrf2^F/F^*, or *Nrf2^LKO^* mice were intraperitoneally injected with vehicle (0.1% dimethylsulfoxide (DMSO) in 90:5:5 of saline, PEG-400, and Tween 80, respectively) or GW7647 (10 mg/kg BW) twice a day (first injection at 00:00 and second injection at 12:00). A total of 5 h after the second injection, mice were sacrificed to collect livers (Figure 1, Figure S1, Figures 4 and 5). To activate NRF2, 8–9 week-old male wild-type C57BL/6J, *Nrf2^F/F^*, or *Nrf2^LKO^* mice were orally gavaged with either vehicle or butylated hydroxyanisole (BHA, 200 mg/kg BW) once a day at 12:00 for 3 days (Figures 4 and 5). To avoid circadian issues, all mice were sacrificed at 17:00–18:00.

### 2.3. Cell Culture and Drug Treatments

AML12 cells were maintained in the complete media: DMEM/F12 high glucose supplemented with 10% FBS, 1% ITS, 1% penicillin-streptomycin antibiotics, and 40 ng/mL dexamethasone. About 70–80% of confluent cells were seeded in a 6- or 12-well plate in a 1:5 ratio 48 h before drug treatments. To assess a pharmacological activity of PPARα in an autophagy-inhibited condition (Figure 1c–e and Figure S2c–e), these cells were treated with either bafilomycin A1 (BafA1) or 3-methyladenine (3-MA) in a dose-dependent manner (BafA1 and 3-MA, 0.1, 0.5, or 1 μM). Simultaneously, they were also treated with synthetic PPARα agonists 1 μM GW7647 or 100 μM Wy-14,643. To determine the pharmacological activity of PPARα in NRF2-activated conditions, AML12 cells were treated with either sulforaphane (SFN, 12.5, 25, or 50 μM) or dimethylfumarate (DMF, 50, 75, or 100 μM) in a dose-dependent manner. Likewise, these cells were also dose-dependently treated with GW7647 (0.01, 0.1, or 1 μM) or Wy-14,643 (1, 10, or 100 μM). On the other hand, AML12 cells were treated with either sulforaphane (25 μM SFN, 6, 12, or 18 h) or dimethylfumarate (75 or 100 μM DMF, 6, 12, or 18 h) in a time-dependent manner. These cells were simultaneously treated with 1 μM GW7647 or 100 μM Wy-14,643 for 24 h. The vehicle is 0.1% DMSO. Mouse embryonic fibroblasts derived from wild-type, *Atg5^−/−^*, or *Atg7^−/−^* mouse embryos were maintained in DMEM high glucose supplemented with 10% FBS, 1% ITS, and 1% penicillin-streptomycin antibiotics.

### 2.4. RNA Purification, cDNA Synthesis, and qPCR Analysis

Total RNA was purified from the snap-frozen mouse liver or AML12 cells using Trizol Reagent according to the manufacturer’s instructions (Invitrogen: Waltham, MA, USA). RNA concentration and quality were determined by Nanodrop. A total of 1 μg of total RNA was converted to cDNA using PrimeScript^TM^ 1st strand cDNA Synthesis kit (Takara Bio Inc.: Kusatsu, Shiga, Japan). qPCR was performed with RbTaq^TM^ qPCR 2X PreMIX (SYBR Green with high ROX) (Enzynomics: Daejeon, Korea) on StepOnePlus Real-Time PCR systems (Applied Biosystems: Waltham, MA, USA). All reactions were carried out in either duplicate or triplicate, and C_t_ values were obtained. mRNA levels were normalized to the expression levels of the housekeeping gene *36b4* (also known as *Rplp0*) using the standard curve method. Information of mouse primer sequences for qPCR analysis is listed in Table S1.

### 2.5. Immunoblot Analysis

~100 mg of frozen liver tissue was homogenized in 1 mL of ice-cold RIPA buffer (25 mM Tris-HCl, pH 7.6, 150 mM NaCl, 1% NP-40, 1% sodium deoxycholate, 1 mM EDTA) supplemented with cOmplete protease and PhosSTOP inhibitors (Roche: Basel, Switzerland), followed by brief sonication in ice. The lysates were centrifuged at 13,000 rpm for 15 min at 4 °C to collect supernatants, of which protein concentration was determined by the Pierce BCA protein assay kit (ThermoFisher Scientific: Waltham, MA, USA). A total of 25 μg of protein lysates were loaded on a 10% SDS-PAGE gel to be resolved, and the proteins were transferred onto an Immuno-Blot PVDF membrane (Bio-Rad Laboratories: Hercules, CA, USA). The membrane was blocked with 5% BSA/TBST (Tris-buffered saline with 0.1% Tween-20) solution for 1 h at room temperature. Primary antibodies were diluted in 5% BSA/TBST solution, which was applied onto the membranes at 4 °C for 16 h. The membrane was washed three times at room temperature (each wash for 15 min; the first and second washes with 5% BSA/TBST solution; the third wash with TBST solution). After washes, the membrane was incubated with an anti-rabbit IgG HRP-linked antibody (Cell Signaling Technology (Danvers, MA, USA)) at room temperature for 1 h, followed by three washes as shown above. The bands were visualized using the Pierce ECL Western Blotting Substrate (ThermoFisher Scientific: Waltham, MA, USA).

### 2.6. Statistical Analysis

All values are shown as mean ± s.e.m. and error bars were derived from biological replicates rather than technical replicates. Significant differences between the two groups were evaluated using a two-tailed, unpaired Student *t*-test, which was found to be appropriate as groups displayed a normal distribution, and comparable variance of *p* < 0.05 was considered statistically significant.

## 3. Results

### 3.1. Autophagy Inhibition Impairs a Pharmacologic PPARα Transactivation

To examine any crosstalk between autophagy and nuclear receptor signaling, liver-specific *Atg7* knockout (*Atg7^LKO^*) mice were generated by crossing male *Alb-Cre* mice with female *Atg7^F/F^* mice. Consistent with previous reports, hepatomegaly phenotypes were observed in *Atg7^LKO^* mice compared with *Atg7^F/F^* control littermates (Figure S1a). As expected, hepatic expression and protein levels of the *Atg7* gene were also markedly reduced in *Atg7^LKO^* mice (Figure S1b,c).

A previous study has shown that increased serum levels of β-hydroxybutyrate, one species of ketone bodies in *Atg7^F/F^* control littermates upon treatment of a synthetic PPARα agonist GW7647 [38], were blunted in *Atg7^LKO^* mice. These results allowed us to set up a hypothesis that core autophagy-related genes are essential for the normal function of PPARα. To test this, hepatic expression levels of PPARα target genes (*Acox1*, *Acot3*, *Fgf21*, *Pdk4*, *Acot1*, and *Cidec*) were determined in fed or fasted *Atg7^F/F^* control littermates and *Atg7^LKO^* mice in response to intraperitoneal treatment of GW7647 (Figure 1a). Consistent with a previous report, pharmacologic inductions of hepatic PPARα target genes were markedly increased in *Atg7^F/F^* mice compared with those of vehicle-treated *Atg7^F/F^* mice. However, these inductions were largely decreased in GW7647-treated *Atg7^LKO^* mice (Figure 1b). To investigate whether these in vivo results recapitulate in cultured cells, we used mouse embryonic fibroblasts (MEFs) derived from wild-type, *Atg5^−/−^*, or *Atg7^−/−^* mice (Figure S2a) [17,39]. As expected, expression levels of *Atg5* or *Atg7* were almost completely absent in either *Atg5^−/−^* or *Atg7^−/−^* MEFs. Treatment of Wy-14,643, a synthetic PPARα agonist in wild-type MEFs robustly increased expressions of PPARα target genes *Acox1*, *Pdk4*, *Acot3*, and *Ucp2*, but these responses were markedly blunted in either *Atg5^−/−^* or *Atg7^−/−^* MEFs (Figure S2b,c). These results suggest that core-autophagy genes are required for the pharmacological PPARα activation.

To examine whether pharmacologic inhibition of autophagy also shows similar results, mouse hepatocyte cell line AML12 cells were dose-dependently treated with 3-methyladenine or bafilomycin A1, well-known autophagy inhibitors with or without synthetic PPARα agonist ligands GW7647 or Wy-14,643 (Figure S2d and Figure 1c) [40,41]. Consistent with in vivo mouse results shown in Figure 1b, inductions of most of PPARα target genes upon GW7647 or Wy-14,643 treatment were dramatically blunted by a dose-dependent treatment of 3-methyladenine or bafilomycin A1 (Figure S2f and Figure 1d,e). All these in vivo and cell culture results indicate that autophagy inhibition can impair a pharmacologic PPARα activity.

Previous reports have also demonstrated that genetic ablations of core autophagy-related genes, including *Atg7*, lead to the accumulation of p62, an autophagy adaptor protein [42]. We could also confirm marked accumulations of p62 in the livers of *Atg7^LKO^* mice (Figure S1d). This causes interactions with KEAP1, an E3 ubiquitin ligase, which liberates NRF2 from the ubiquitin-mediated proteasomal degradation system. Free cytoplasmic NRF2 is then translocated into the nucleus, where it acts as an antioxidant transcription factor to remove reactive oxygen species [21]. As expected, we also observed dramatically increased expression levels of the *Nqo1* gene, an NRF2 target gene in the livers of *Atg7^LKO^* mice compared with those of *Atg7^F/F^* mice (Figure S1e). *Atg5^−/−^* and *Atg7^−/−^* MEFs also increased *Nqo1* and *Hmox1* expressions (Figure S2a). Based on these data, we concluded that our *Atg7^LKO^* mice also increased hepatic NRF2 activation. It is of interest to note that the pharmacologic PPARα activation by GW7647 treatment does not significantly alter hepatic *Nqo1* expression compared with vehicle-treated counterparts (Figure S1e). We also found that a dose-dependent treatment of bafilomycin A1 in AML12 cells markedly increased expression levels of the *Hmox1* gene, another known NRF2 target gene. However, expression levels of the *Nqo1* gene showed opposite results, which might reflect different expression kinetics of two known NRF2 target genes in response to autophagy inhibition (Figure S1f,g). Treatment of 3-methyladenine in AML12 cells showed similar results (Figure S2d,e).

### 3.2. SFN-Mediated NRF2 Activation in a Dose-Dependent Manner Impairs a Pharmacologic PPARα Transactivation

**Figure 2:**
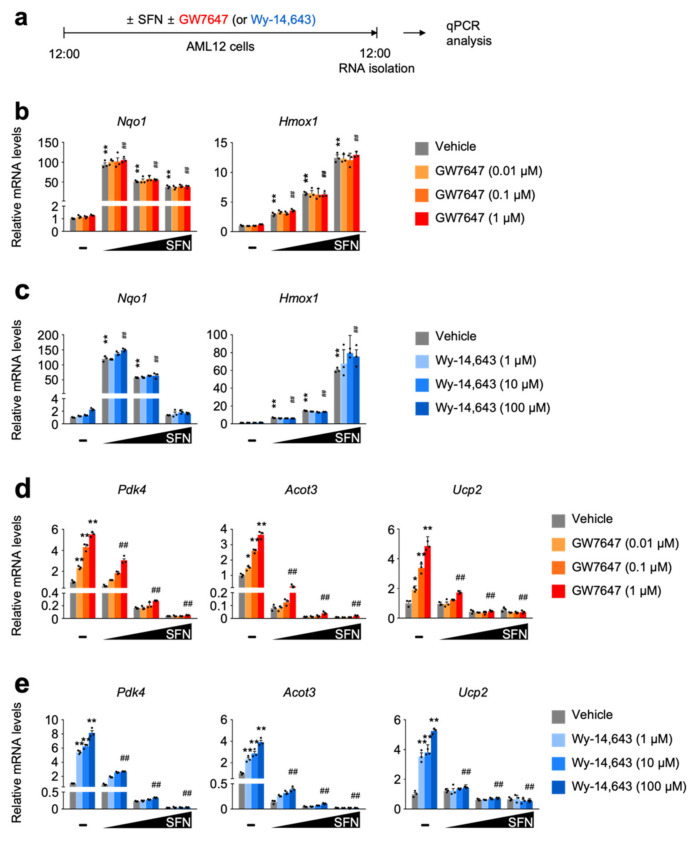
SFN-mediated NRF2 activation in a dose-dependent manner impairs pharmacologic PPAR𝛼 transactivation. (**a**) A schematic diagram of an experimental procedure in AML12 cells. AML12 cells were treated with sulforaphane (SFN, 12.5, 25, or 50 μM), a known NRF2 activator in a dose-dependent manner in the absence or presence of synthetic PPAR𝛼 agonists (GW7647, 0.01, 0.1 or 1 μM; Wy-14,643, 1, 10 or 100 μM) for 24 h. Vehicle is 0.1% DMSO. Total RNAs from these cells were prepared to perform qPCR analysis. (**b**,**c**) Expression levels of NRF2 target genes *Nqo1* and *Hmox1* were determined in AML12 cells shown in panel (**a**) by qPCR analysis. *n* = 3 per group, ** *p* < 0.01 vs. AML12 cells treated with vehicle. Data represent mean ± s.e.m. and are plotted as fold change. Each dot indicates an individual well. (**d**,**e**) Expression levels of PPAR𝛼 target genes *Pdk4*, *Acot3*, and *Ucp2,* were determined in AML12 cells shown in panel (**a**) by qPCR analysis. *n* = 3 per group, ** *p* < 0.01 vs. AML12 cells treated with vehicle. ^##^ *p* < 0.01 vs. AML12 cells treated with 1 μM GW7647 or 100 μM Wy-14,643. Data represent mean ± s.e.m. and are plotted as fold change. Each dot indicates an individual sample. Statistics by a two-tailed, unpaired Student *t*-test.

To examine whether NRF2 activation directly affects PPARα activity, AML12 cells were dose-dependently treated with sulforaphane [43] or dimethylfumarate [44], potent NRF2 activators in the presence or absence of synthetic PPARα agonist ligands GW7647 or Wy-14,643 (Figure 2a and Figure S2a). As expected, sulforaphane or dimethylfumarate dramatically increases the expression of NRF2 target genes *Nqo1* and *Hmox1*, which are not altered by the treatment of synthetic PPARα agonists (Figure 2b,c and Figure S2b,c). These data indicate that a pharmacologic PPARα activation may not affect sulforaphane or dimethylfumarate-mediated NRF2 activation.

Intriguingly, however, completely opposite results were observed in terms of a pharmacologic PPARα activation in the presence of NRF2 activators. We found that dose-dependent treatments of sulforaphane or dimethylfumarate markedly downregulated expression of PPARα target genes *Pdk4*, *Acot3*, and *Ucp2* in response to GW7647 or Wy-14,643 treatment (Figure 2d,e and Figure S2d,e). All these data suggest that NRF2 activation might mediate the suppression of a pharmacologic PPARα activation.

### 3.3. SFN-Mediated NRF2 Activation in a Time-Dependent Manner Impairs a Pharmacologic PPARα Transactivation

**Figure 3:**
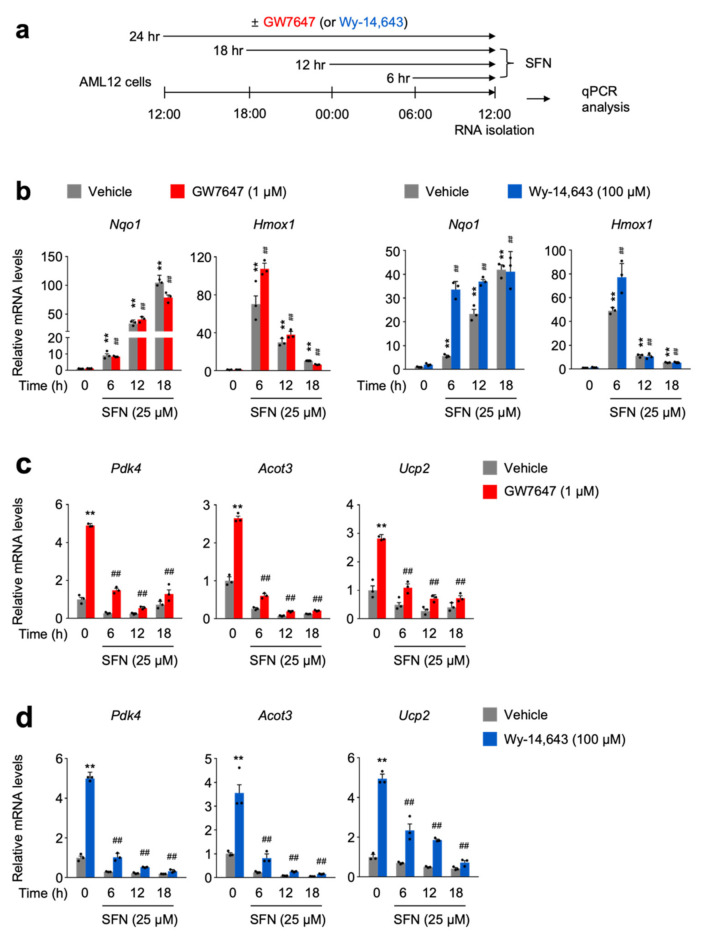
SFN-mediated NRF2 activation in a time-dependent manner impairs pharmacologic PPAR𝛼 transactivation. (**a**) A schematic diagram of an experimental procedure in AML12 cells. AML12 cells were treated with 25 μM of SFN, a known NRF2 activator in a time-dependent manner (6, 12, or 18 h) in the absence or presence of synthetic PPAR𝛼 agonists (GW7647, 0.01, 0.1 or 1 μM; Wy-14,643, 1, 10 or 100 μM) for 24 h. Vehicle is 0.1% DMSO. Total RNAs from these cells were prepared to perform qPCR analysis. (**b**,**c**) Expression levels of NRF2 target genes *Nqo1* and *Hmox1* were determined in AML12 cells shown in panel (**a**) by qPCR analysis. (**d**) Expression levels of PPAR𝛼 target genes *Pdk4*, *Acot3*, and *Ucp2,* were determined in AML12 cells shown in panel (**a**) by qPCR analysis. *n* = 3 per group, ** *p* < 0.01 vs. AML12 cells treated with vehicle. ^##^ *p* < 0.01 vs. AML12 cells treated with GW7647 or Wy-14,643. Data represent mean ± s.e.m. and are plotted as fold change. Each dot indicates an individual sample. Statistics by a two-tailed, unpaired Student *t*-test.

To further thoroughly investigate whether NRF2 activation directly affects PPARα activity, AML12 cells were time-dependently treated with sulforaphane or dimethylfumarate in the presence or absence of GW7647 or Wy-14,643 (Figure 3a and Figure S3a). As expected, these NRF2 activators dramatically increase expression of the *Nqo1* gene in a time-dependent manner (Figure 3b and Figure S3b). However, as mentioned above, we again observed different expression kinetics of two known NRF2 target genes, *Nqo1* and *Hmox1*. A total of 6 h treatment of NRF2 activators showed the highest expression levels of *Hmox1* gene whose mRNA levels were gradually decreased by further increasing treatment time of NRF2 activators (Figure 3b and Figure S3b). Nevertheless, time-dependent treatments of sulforaphane or dimethylfumarate in AML12 cells dramatically suppress the expression of PPARα target genes *Pdk4*, *Acot3*, and *Ucp2* in response to GW7647 or Wy-14,643 (Figure 3c,d and Figure S3c,d). All these data support that acute NRF2 activation might mediate the suppression of the pharmacologic PPARα activation.

### 3.4. NRF2 Activation Is Necessary and Sufficient for Suppressing a Pharmacologic PPARα Transactivation

**Figure 4:**
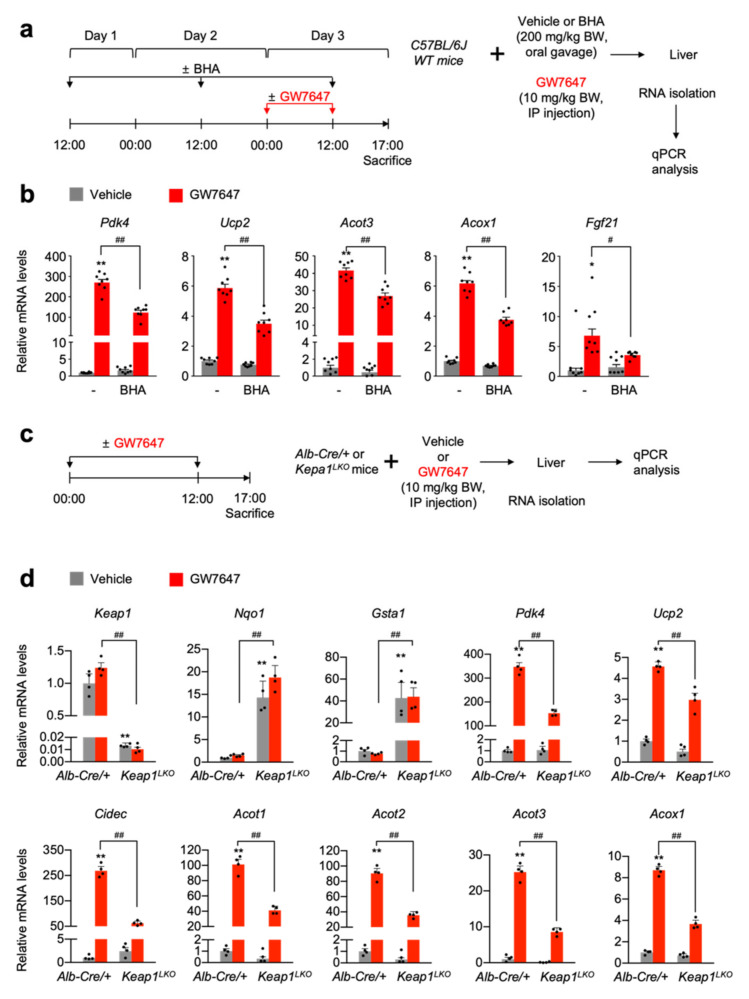
NRF2 activation impairs pharmacologic PPAR𝛼 transactivation in vivo. (**a**) A schematic diagram of an experimental procedure in mice. The 8- to 9-week-old male C57BL/6J wild-type mice were orally gavaged with vehicle or butylated hydroxyanisole (BHA, 200 mg/kg BW) once a day for 3 days. During the last 24 h, all mice were intraperitoneally injected with vehicle (0.1% DMSO in 4:1 ratio of PEG 400 and Tween 80) or GW7647, a synthetic PPAR𝛼 agonist (10 mg/kg BW) twice a day (first injection: 00:00 am, second injection: 12:00 pm). A total of 5 h after last treatment, all mice were killed to collect livers of which total RNAs were prepared for qPCR analysis. (**b**) Hepatic expression levels of PPAR𝛼 target genes *Pdk4*, *Upc2*, *Acot3*, *Acox1*, and *Fgf21* were determined as shown in panel (**a**) by qPCR analysis. *n* = 8 per group, * p < 0.05, ** p < 0.01 vs. WT treated with vehicle. (**c**) A schematic diagram of an experimental procedure in mice. The 8- to 9-week-old male *Alb-Cre/+* and *Keap1^LKO^* mice were intraperitoneally injected with vehicle (0.1% DMSO in 4:1 ratio of PEG 400 and Tween 80) or GW7647 (10 mg/kg BW) twice a day. A total of 5 h after last treatment, all mice were killed to collect livers, of which total RNAs were isolated for qPCR analysis. (**d**) Hepatic expression levels of *Keap1*, NRF2 target genes (*Nqo1* and *Gsta1*), and PPAR𝛼 target genes *Pdk4*, *Upc2*, *Cidec*, *Acot1* to *3*, and *Acox1* were determined shown in panel (**c**) by qPCR analysis. *n* = 4 per group, ** p < 0.01 vs. *Alb-Cre/+* treated with vehicle. *^#^ p* < 0.05, *^##^ p* < 0.01. Data represent mean ± s.e.m. and are plotted as fold change. Each dot indicates an individual mouse. Statistics by a two-tailed, unpaired Student *t*-test. WT, wild-type.

To further confirm these results in vivo, C57BL/6J wild-type mice were orally gavaged with butylated hydroxyanisole (BHA), an NRF2 activator, for 3 days [45]. On day 3, these mice were intraperitoneally treated with vehicle or GW7647, as shown in Figure 4a. After that, hepatic expression levels of PPARα target genes *Pdk4*, *Ucp2*, *Acot3*, *Acox1*, and *Fgf21* were determined. As expected, GW7647 treatment markedly increased mRNA levels of these PPARα target genes, but these responses were significantly blunted in the livers of BHA-treated mice (Figure 4b). These data indicate that BHA-mediated NRF2 activation is able to suppress a pharmacologic PPARα activation.

To genetically validate these results, we generated liver-specific *Keap1* knockout (*Keap1^LKO^*) mice by crossing male *Alb-Cre* mice with female *Keap1^F/F^* mice. Since floxed *Keap1* alleles in *Keap1^F/F^* mice turned out to be hypomorphic, leading to the spontaneous activation of NRF2, we used *Alb-Cre* control littermates as control mice instead of *Keap1^F/F^* mice (Figure 4c) [46]. As expected, *Keap1^LKO^* mice showed almost an absence of hepatic *Keap1* expressions but marked increased mRNA levels of *Nqo1* and *Gasta1,* indicating that a potent NRF2 activation occurs in the livers of *Keap1^LKO^* mice. Once again, hepatic expression levels of PPARα target genes *Pdk4*, *Ucp2*, *Cidec*, *Acot1 to 3*, and *Acox1* were significantly downregulated in GW7647-treated *Keap1^LKO^* mice (Figure 4d). These data suggest that genetic NRF2 activation leads to the inhibition of the pharmacological PPARα activation.

**Figure 5:**
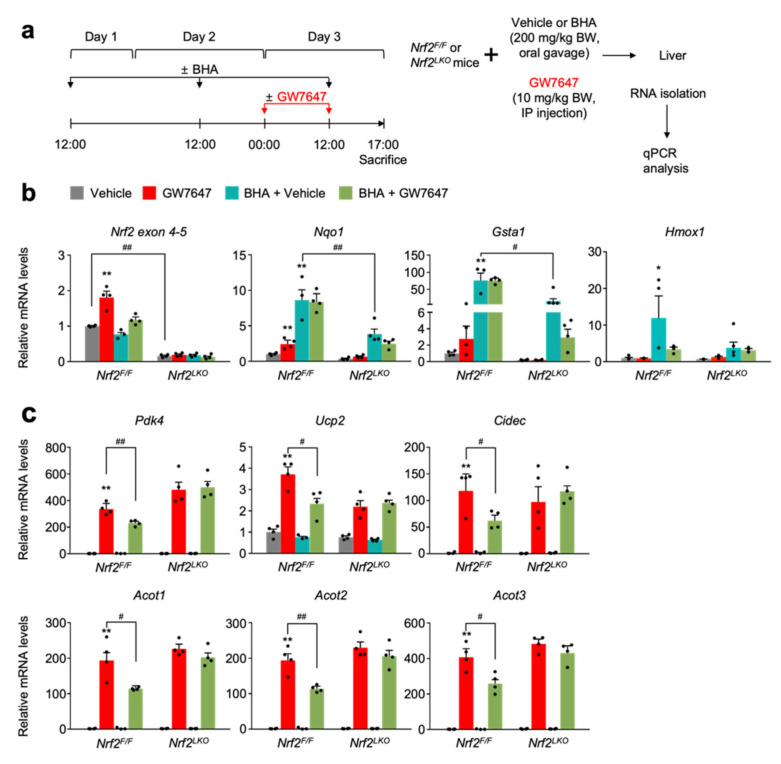
NRF2 activation is necessary and sufficient for suppressing pharmacologic PPAR𝛼 transactivation. (**a**) A schematic diagram of an experimental procedure in mice. The 8- to 9-week-old male *Nrf2^F/F^* and *Nrf2^LKO^* mice were orally gavaged with vehicle or butylated hydroxyanisole (BHA, 200 mg/kg BW) once a day for 3 days. During the last 24 h, all mice were intraperitoneally injected with vehicle (0.1% DMSO in 4:1 ratio of PEG 400 and Tween 80) or GW7647, a synthetic PPAR𝛼 agonist (10 mg/kg BW) twice a day (first injection: 00:00 am, second injection: 12:00 pm). A total of 5 h after last treatment, all mice were killed to collect livers of which total RNAs were prepared for qPCR analysis. (**b**) Hepatic expression levels of *Nrf2* and its target genes *Nqo1*, *Gsta1*, and *Hmox1* were determined in AML12 cells shown in panel (**a**) by qPCR analysis, * *p* < 0.05. (**c**) Hepatic expression levels of PPAR𝛼 target genes *Pdk4*, *Upc2*, *Cidec*, and *Acot1* to *3* were determined as shown in panel (**a**) by qPCR analysis. *n* = 3–4 per group, ** *p* < 0.01 vs. *Nrf2^F/F^* treated with vehicle. ^#^ *p* < 0.05, ^##^ *p* < 0.01. Data represent mean ± s.e.m. and are plotted as fold change. Each dot indicates an individual mouse. Statistics by a two-tailed, unpaired Student *t*-test.

To genetically validate whether these blunted pharmacological PPARα activations mediated by NRF2 activators depend on NRF2 proteins, we first generated liver-specific Nrf2 knockout (*Nrf2^LKO^*) mice by crossing male *Alb-Cre* mice with female *Nrf2^F/F^* mice. *Nrf2^F/F^* control littermates and *Nrf2^LKO^* mice were then orally gavaged with BHA for 3 days. On day 3, these mice were intraperitoneally treated with vehicle or GW7647, as shown in Figure 5a. As expected, hepatic *Nrf2* mRNA levels were markedly reduced in *Nrf2^LKO^* mice, confirming a genetic ablation of the *Nrf2* gene in the liver (Figure 5b). Like the results obtained in C57BL/6J mice (Figure 4), BHA treatment also robustly increased hepatic expression levels of NRF2 target genes *Nqo1* and *Gasta1* in *Nrf2^F/F^* control littermates, but these responses were significantly blunted in *Nrf2^LKO^* mice (Figure 5b).

These results demonstrate that induction of *Nqo1* and *Gsta1* genes by BHA treatment largely depends on NRF2. Moreover, GW7647 treatment in *Nrf2^F/F^* control littermates also robustly induced hepatic expressions of PPAR target genes *Pdk4, Ucp2, Cidec,* and *Acot1 to 3,* but these inductions were significantly blunted in BHA-treated *Nrf2^F/F^* counterparts. However, these suppressed expression levels of PPAR target genes were almost completely lost in BHA-treated *Nrf2^LKO^* mice, demonstrating that blunted pharmacological transactivation of PPARα mediated by BHA treatment indeed requires NRF2 proteins (Figure 5c).

## 4. Discussion

In this study, we showed that inhibition of basal autophagy impairs a pharmacological activation of the fasting-activated nuclear receptor PPARα. Moreover, our data also suggested that NRF2 known to be activated in autophagy-defective conditions might mediate this effect. In the clinical aspects, this phenomenon could be critical, in particular for hyperlipidemia patients taking fibrate derivatives. Simultaneous activation of both NRF2 and PPARα transcription factors might diminish the beneficial effects of fibrate medications.

Similar results have also been reported by the Komatsu and Panasyuk laboratories in regards to the impairment of PPARα transactivation by autophagy defects, although their molecular mechanisms are distinct from ours [47,48]. They have found that NCoR1 corepressor proteins are remarkedly accumulated in autophagy-defective hepatocytes obtained from *Atg5*, *Atg7*, or *Vps15* conditional knockout mice. These increased levels of NCoR1 tend to be recruited to PPARα, which leads to suppression of its target gene expression. In terms of the detailed molecular mechanism by which autophagy defects impede PPARα signaling, we speculate that there are many more mechanisms yet to be discovered.

Similar to our findings, sulforaphane treatment in 3T3-L1 cells inhibits adipocyte differentiation by reducing the expression of PPARγ, a master regulator of adipogenesis, indicating that NRF2 activation might impair functionalities of other PPARs [30]. Conversely, NRF2 activation also increases the expression of PPARs. Treatment of either BHA or sulforaphane has been shown to induce hepatic PPARγ or PPARβ/δ expression in livers of wild-type mice [31,32]. Consistently, in our experiments in Figure 4a, we could confirm that a 3-day treatment of BHA increases expression of both *Pparγ* and *Pparβ/δ* genes (data not shown). Moreover, cooperative actions between PPARγ and NRF2 have also been reported. In oxidative stress conditions, both transcription factors reciprocally induce their expression as an upstream regulator, resulting in increased expressions of many cytoprotective genes encoding HO-1, CAT, SOD, and GST. These enzymes have been known to alleviate reactive oxygen species in cells and tissues [33,34].

It has been known that heme oxygenase-1 (HO-1) encoded by the *Hmox1* gene, a known NRF2 target gene, produces biliverdin from heme, and biliverdin reductase then converts biliverdin to bilirubin [49]. Interestingly, the Hinds laboratory has shown that bilirubin might act as an endogenous agonist ligand for PPARα, although its physiological relevance to the fasting-activated PPARα signaling should be confirmed [50,51]. According to this scenario, PPARα target genes might be induced rather than reduced via an HO-1/bilirubin/PPARα axis. However, we found that treatment of sulforaphane, dimethylfumarate, or BHA, or liver-specific *Keap1* deletion did not increase basal expression levels of most PPARα target genes at least we examined, indicating that NRF2 activation might not affect a basal activity of PPARα.

In the future, the generation of liver-specific *Atg7* and *Nrf2* double knockout (*Atg7^LKO^*; *Nrf2^LKO^*) mice may provide a valuable resource to address whether NRF2 activation in *Atg7^LKO^* mice is genetically required for suppressing the pharmacological activation of the nuclear receptor PPARα. Moreover, the generation of liver-specific *Nrf2* and *Keap1* double knockout (*Nrf2^LKO^*; *Keap1^LKO^*) mice may also give us important insights to examine whether the blunted pharmacological activation of PPARα in *Keap1^LKO^* mice is genetically dependent on NRF2.

Further investigations are also necessary to define detailed molecular mechanisms by which nuclear NRF2 proteins interfere with the functions of the nuclear receptor PPARα. This could be achieved in a variety of mechanisms, including a direct protein-protein interaction, squelching coactivators, interfering with the recruitment of coactivators, promoting recruitment of corepressors, inhibiting nuclear receptor binding to its response element of target genes, and so on.

## 5. Conclusions

**Figure 6:**
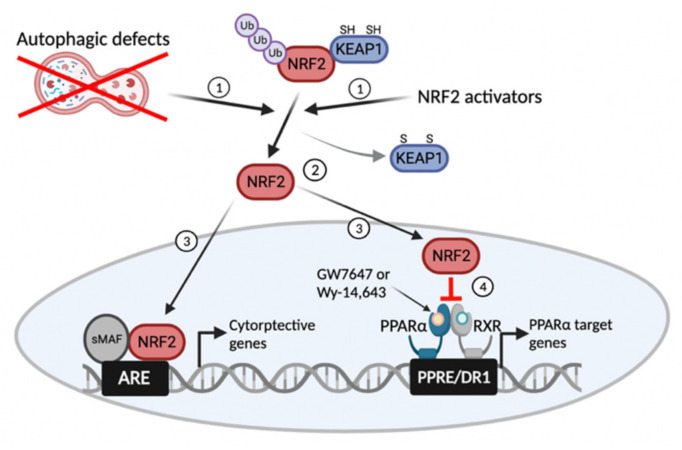
Working model of basal autophagy defects or NRF2 activators that impair pharmacological transactivation of the nuclear receptor PPARα. Similar to NRF2 activators, autophagy inhibition by pharmacologic or genetic manipulations liberates NRF2 from KEAP1-mediated proteasomal degradation (1, 2). This event leads to the nuclear translocation of NRF2 from the cytoplasm, which acts as a potent antioxidant transcription factor regulating the expression of many cytoprotective genes (3). On the other hand, the nuclear NRF2 interferes with a pharmacologic nuclear receptor signaling, including PPARα (4). ARE, antioxidant response element; PPRE, peroxisome proliferator response element; DR1, direct repeat 1. This schematic diagram was created in BioRender.com (accessed on 6 January 2022).

In summary, we propose an essential role of basal autophagy activity for the pharmacological functions of the nuclear receptor PPARα. Autophagic defects lead to NRF2 translocation from the cytoplasm to the nucleus, where it acts as an antioxidant transcription factor by regulating many cytoprotective genes. We speculate that these nuclear NRF2 transcription factor proteins could interfere with the activities of many other transcription factors, including PPARα, via a variety of mechanisms (Figure 6). This study establishes the unexpected role of basal autophagy for the therapeutic effects of pharmacologically targeting the nuclear receptor PPARα, which may be useful for the treatment of diverse metabolic diseases.

## Data Availability

The data presented in this study are available on request from the corresponding author.

## Supplementary Materials

The following supporting information can be downloaded at: https://www.mdpi.com/2073-4409/11/4/754/s1, Figure S1: Upregulation of NRF2 target genes in response to autophagy inhibition; Figure S2: Both genetic and pharmacological inhibitions of autophagy suppress the pharmacological PPARα activation. Figure S3: DMF-mediated NRF2 activation in a dose-dependent manner impairs a pharmacologic PPARα transactivation; Figure S4: DMF-mediated NRF2 activation in a time-dependent manner impairs a pharmacologic PPARα transactivation; Table S1: Mouse primer sequences for qPCR analysis.
