# Supplementary material for: Basal Autophagy Is Necessary for A Pharmacologic PPARα Transactivation
Source: Cells. 2022 Feb 21;11(4):754. doi: 10.3390/cells11040754 (PMC8870620; doi:10.3390/cells11040754)
Supplement: Supplementary file 1 [file cells-11-00754-s001.zip › Figure S1 to S4_Table S1.pdf]

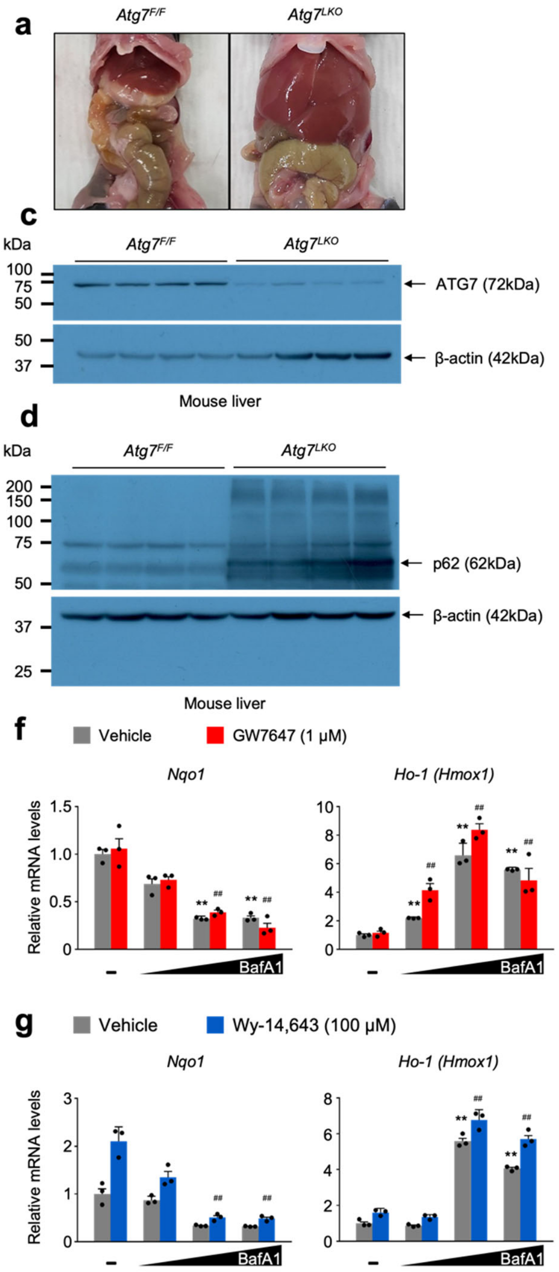


**Figure S1.** Upregulation of NRF2 target genes in response to autophagy inhibition. Male *Alb-Cre* mice were crossed with female *Atg7*$^{F/F}$ mice to generate liver-specific *Atg7* knockout (*Atg7*$^{LKO}$) mice as well as control littermate *Atg7*$^{F/F}$ mice. (**a**) Hepatomegaly was confirmed in *Atg7*$^{LKO}$ mice. (**b**) Hepatic expression levels of *Atg7* gene were determined in *Atg7*$^{fF/fF}$ and *Atg7*$^{LKO}$ mice by qPCR analysis. n = 3 per group, **$p < 0.01$ vs *Atg7*$^{LKO}$ mice. Data represent mean ± s.e.m. and are plotted as fold change. (**c**-**d**) Hepatic ATG7 and p62 protein levels were assessed in *Atg7*$^{F/F}$ and *Atg7*$^{LKO}$ mice by immunoblot analysis. β-actin is a loading control. Each lane indicates individual mouse. (**e**) Hepatic expression levels of *Nqo1*, an NRF2 target gene were determined in *Atg7*$^{F/F}$ and *Atg7*$^{LKO}$ mice shown in Figure 1a by qPCR analysis. n = 4 per group, **$p < 0.01$ vs *Atg7*$^{F/F}$ mice treated with vehicle. $^{\#\#}p < 0.01$. Data represent mean ± s.e.m. and are plotted as fold change. Each dot indicates individual mouse. (**f**,**g**) Expression levels of NRF2 target genes *Nqo1* and *Hmox1* were determined in AML12 cells shown in Figure 1c by qPCR analysis. n = 3 per group, **$p < 0.01$ vs AML12 cells treated with vehicle. $^{\#\#}p < 0.01$ vs AML12 cells treated with GW7647. Data represent mean ± s.e.m. and are plotted as fold change. Each dot indicates individual sample. Statistics by a two-tailed, unpaired Student *t*-test.

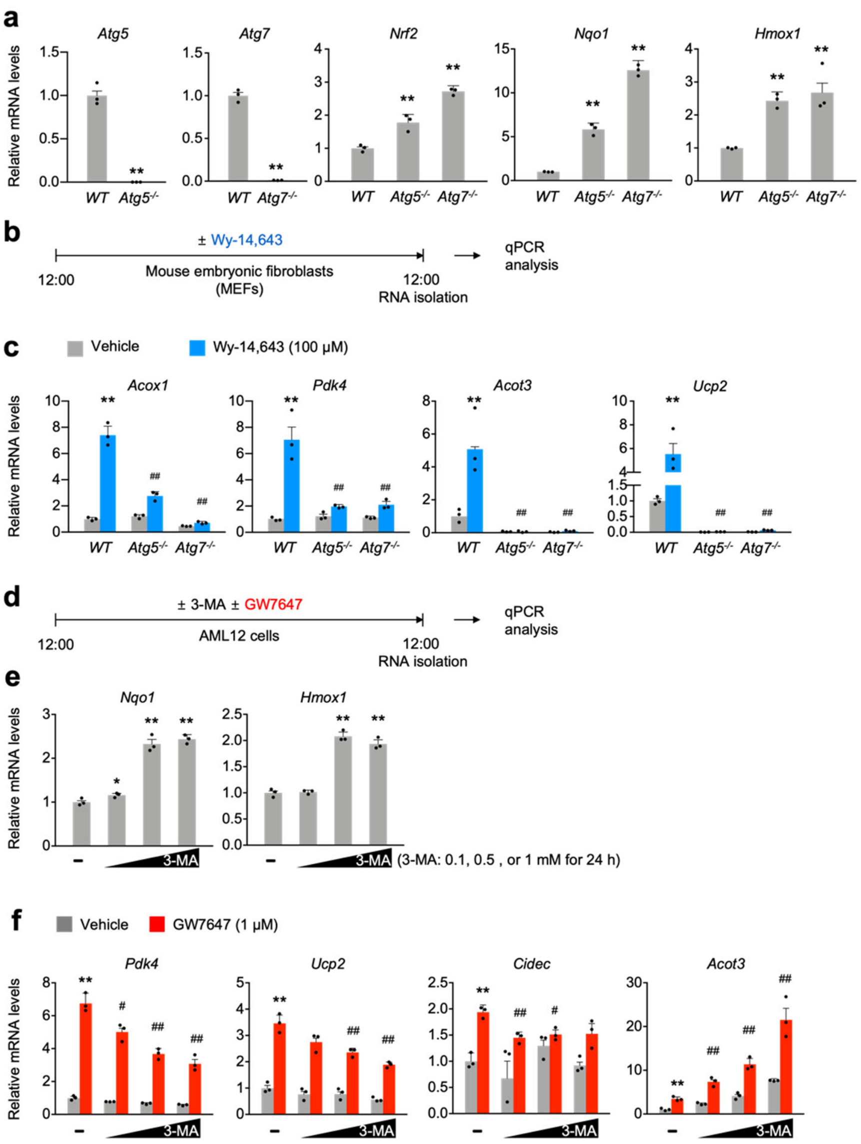


**Figure S2.** Both genetic and pharmacological inhibitions of autophagy suppress the pharmacological PPARα activation. (**a**) Expression levels of core-autophagy genes *Atg5* and *Atg7*, and NRF2 target genes *Nrf2*, *Nqo1*, and *Hmox1* were determined in wild-type, $Atg5^{-/-}$, or $Atg7^{-/-}$ mouse embryonic fibroblasts (MEFs) by qPCR analysis. n = 3 per group, ***p* < 0.01 vs WT MEFs. (**b**) A schematic diagram of an experimental procedure in MEFs. These cells treated with either vehicle or Wy-14,643 (100 μM), the synthetic PPAR*α* agonist for 24 h. Vehicle is 0.1% DMSO. Total RNAs from these cells were prepared to perform qPCR analysis. (**c**) Expression levels of PPARα target genes *Acox1*, *Pdk4*, *Acot3*, and *Ucp2* were determined in MEFs shown in panel **a** by qPCR analysis. n = 3 per group. ***p* < 0.01 vs. WT MEFs treated with vehicle. $^{\#\#}P < 0.01$ vs. WT MEFs treated with Wy-14,643. (**d**) A schematic diagram of an experimental procedure in AML12 cells. AML12 cells were treated with 3-methyladenine (3-MA, 0.1, 0.5, or 1 mM), an autophagy inhibitor in a dose-dependent manner in the absence or presence of GW7647 (1 μM) for 24 hr. Vehicle is 0.1% DMSO. Total RNAs from these cells were prepared to perform qPCR analysis. (**e**) Expression levels of NRF2 target genes *Nqo1*, and *Hmox1* were determined in AML12 cells shown in panel **d** by qPCR analysis. n = 3 per group, ***p* < 0.01 vs AML12 cells treated with vehicle (denoted by "-"). (**f**) Expression levels of PPARα target genes *Pdk4*, *Ucp2*, *Cidec*, and *Acot3* were determined in AML12 cells shown in panel **d** by qPCR analysis. n = 3 per group. ***p* < 0.01 vs. AML12 cells treated with vehicle. $^{\#\#}P < 0.01$ vs. AML12 cells treated with GW7647. Data represent mean ± s.e.m. and are plotted as fold change. Each dot indicates individual well. Statistics by a two-tailed, unpaired Student *t*-test. WT, wild-type.

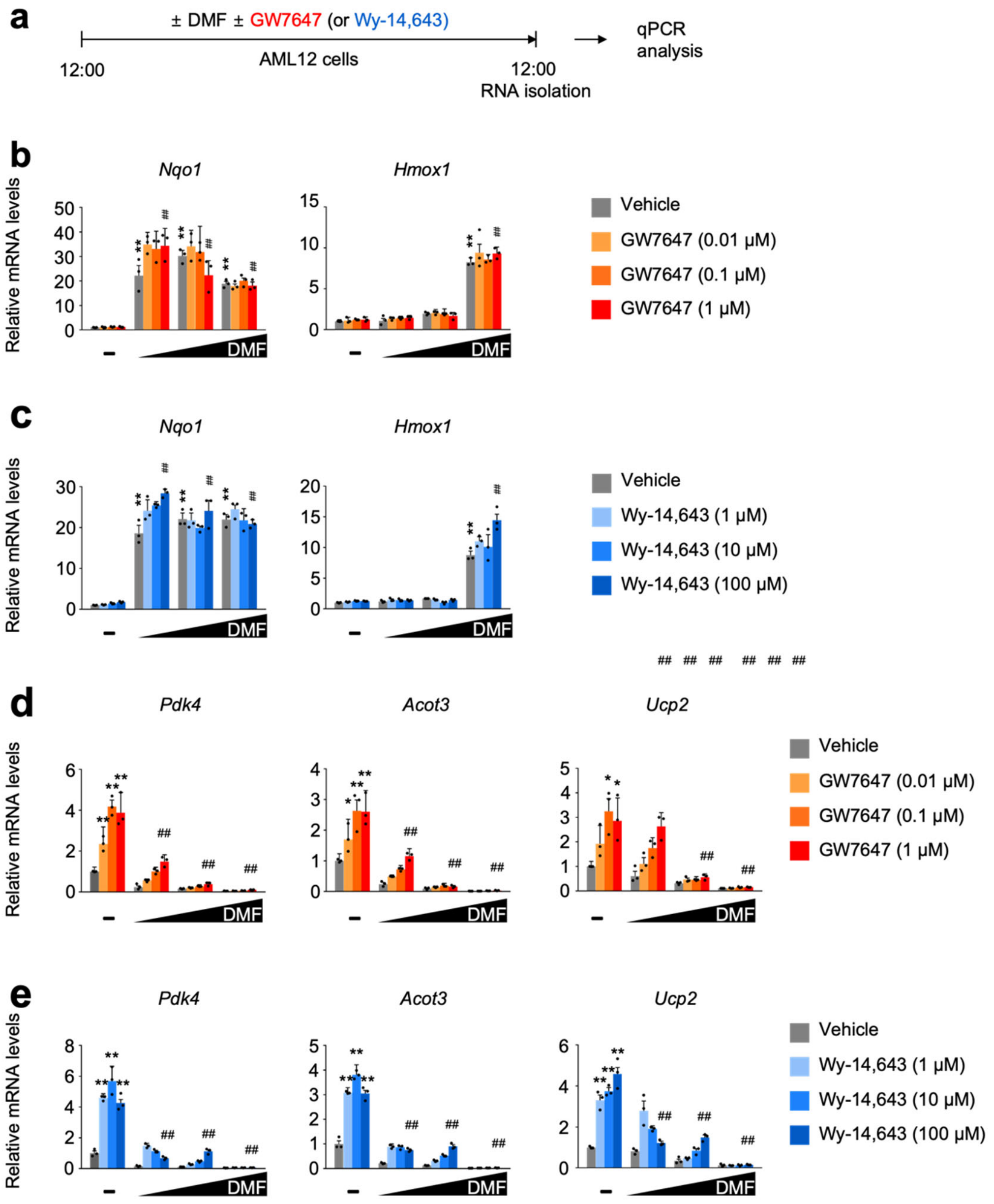


**Figure S3**. DMF-mediated NRF2 activation in a dose-dependent manner impairs a pharmacologic PPAR*α* transactivation. (**a**) A schematic diagram of an experimental procedure in AML12 cells. AML12 cells were treated with dimethylfumarate (DMF, 50, 75, or 100 μM), a known NRF2 activator in a dose-dependent manner in the absence or presence of synthetic PPAR*α* agonists (GW7647: 0.01, 0.1 or 1 μM; Wy-14,643: 1, 10 or 100 μM) for 24 hr. Vehicle is 0.1% DMSO. Total RNAs from these cells were prepared to perform qPCR analysis. (**b**,**c**) Expression levels of NRF2 target genes *Nqo1* and *Hmox1* were determined in AML12 cells shown in panel **a** by qPCR analysis. n = 3 per group. Data represent mean ± s.e.m. and are plotted as fold change. Each dot indicates individual well. $^{**}p < 0.01$ vs. AML12 cells treated with vehicle. $^{\#\#}p < 0.01$ vs. AML12 cells treated with 1 μM GW7647. (**d**,**e**), Expression levels of PPAR*α* target genes *Pdk4*, *Acot3*, and *Ucp2* were determined in AML12 cells shown in panel **a** by qPCR analysis. n = 3 per group, $^{*}p < 0.05$, $^{**}p < 0.01$ vs. AML12 cells treated with vehicle. $^{\#\#}p < 0.01$ vs. AML12 cells treated with either 1 μM GW7647 or 100 μM Wy-14,643. Data represent mean ± s.e.m. and are plotted as fold change. Each dot indicates individual sample. Statistics by a two-tailed, unpaired Student *t*-test (**d**,**e**).

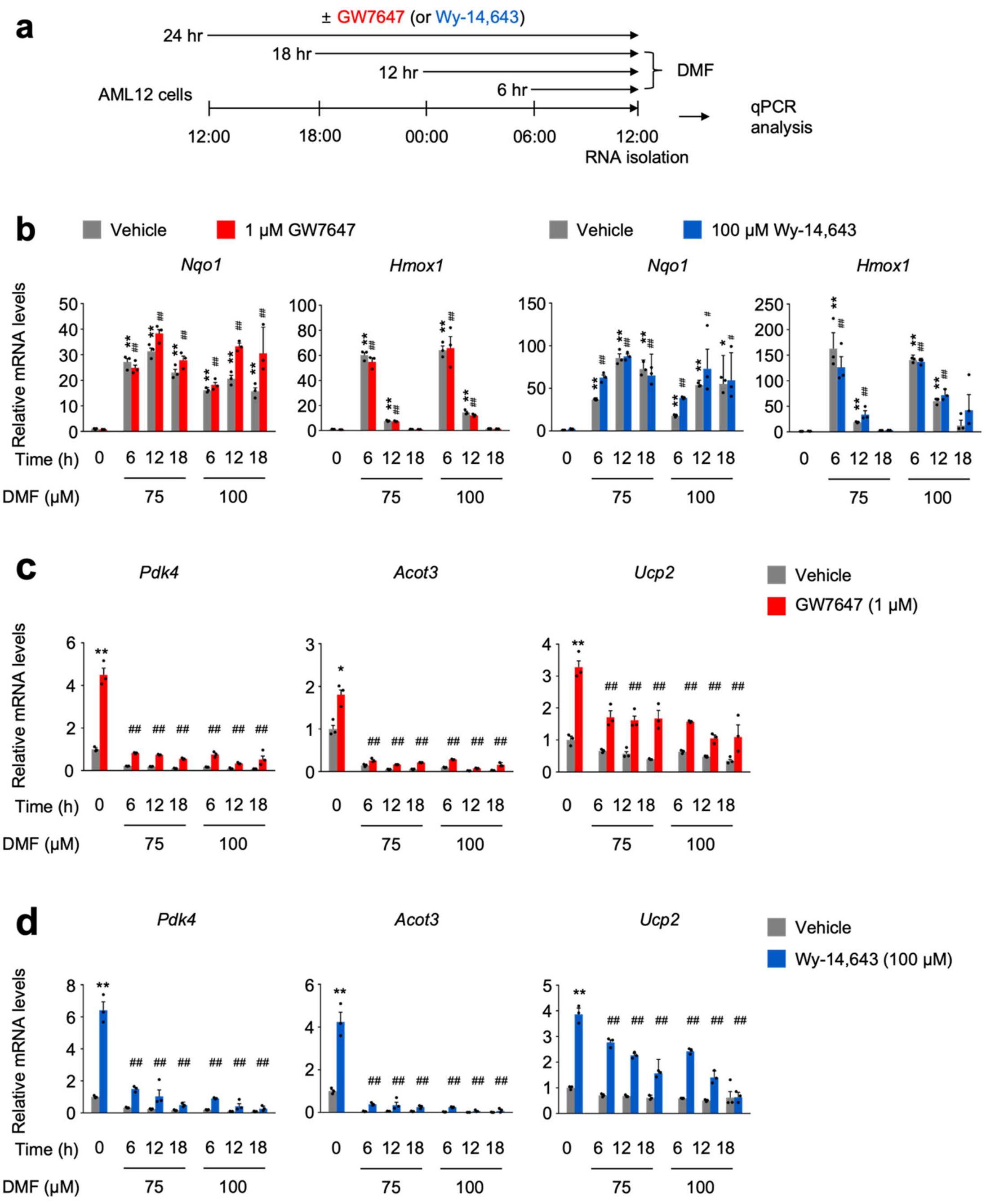


**Figure S4**. DMF-mediated NRF2 activation in a time-dependent manner impairs a pharmacologic PPAR*α* transactivation. (**a**) A schematic diagram of an experimental procedure in AML12 cells. AML12 cells were treated with either 75 μM or 100 μM of dimethylfumarate (DMF), a known NRF2 activator in a time-dependent manner (6 h, 12 h or 18 h) in the absence or presence of synthetic PPAR*α* agonists (1 μM of GW7647; 100 μM of Wy-14,643) for 24 h. Vehicle is 0.1% DMSO. Total RNAs from these cells were prepared to perform qPCR analysis. (**b**) Expression levels of NRF2 target genes *Nqo1* and *Hmox1* were determined in AML12 cells shown in panel **a** by qPCR analysis. **p* < 0.05, ***p* < 0.01 vs. AML12 cells treated with vehicle. ##*p* < 0.01 vs. AML12 cells treated with either GW7647 or Wy-14,643. (**c**,**d**) Expression levels of PPAR*α* target genes *Pdk4*, *Acot3*, and *Ucp2* were determined in AML12 cells shown in panel **a** by qPCR analysis. n = 3 per group, **p* < 0.05, ***p* < 0.01 vs. AML12 cells treated with vehicle. ##p < 0.01 vs. AML12 cells treated with either GW7647 or Wy-14,643. Data represent mean ± s.e.m. and are plotted as fold change. Each dot indicates individual sample. Statistics by a two-tailed, unpaired Student *t*-test.

**Table S1**. Mouse primer sequences for qPCR analysis

| Gene | Sequences |
|---|---|
| Atg7 exon 14-15 Fwd | CTAATGGACACCAGGGAGAG |
| Atg7 exon 14-15 Rvs | CATGTCTCATGACAACAAAGGT |
| Pdk4 Fwd | GTCGAGCATCAAGAAAACCGTCC |
| Pdk4 Rvs | TGTGATGCCCTTCAGGAAGGAG |
| Acot3 Fwd | ACTACGAGGACCTCCCTAAGGA |
| Acot3 Rvs | CATGGCAAAGCCAAGTTCACCC |
| Ucp2 Fwd | TAAAGGTCCGCTTCCAGGCTCA |
| Ucp2 Rvs | ACGGGCAACATTGGGAGAAGTC |
| Acot2 Fwd | AAGAAGCCGTGAACTACCTGCG |
| Acot2 Rvs | TGTGATGCCCTTCAGGAAGGAG |
| Cidec Fwd | TCGGAAGGTTCGCAAAGGCATC |
| Cidec Rvs | CTCCACGATTGTGCCATCTTCC |
| Acox1 Fwd | GCCATTCGATACAGTGCTGTGAG |
| Acox1 Rvs | CCGAGAAAGTGGAAGGCATAGG |
| Fgf21 Fwd | ATCAGGGAGGATGGAACAGTGG |
| Fgf21 Rvs | AGCTCCATCTGGCTGTTGGCAA |
| Nrf2 exon 4-5 Fwd | TTAAGCAGCATAGAGCAGGA |
| Nrf2 exon 4-5 Rvs | TTCTGTCAGTGTGGCTTCTG |
| Nqo1 Fwd | GCCGAACACAAGAAGCTGGAAG |
| Nqo1 Rvs | GGCAAATCCTGCTACGAGCACT |
| Hmox-1 Fwd | CACTCTGGAGATGACACCTGAG |
| Hmox-1 Rvs | GTGTTCCTCTGTCAGCATCACC |
| Gstp1 Fwd | TGGAAGGAGGAGGTGGTTACCA |
| Gstp1 Rvs | GGTAAAGGGTGAGGTCTCCATC |
| 36B4 Fwd | CAACCCAGCTCTGGAGAAAC |
| 36B4 Rvs | CCAACAGCATATCCCGAATC |
| Keap1 exon 4-5 Fwd | ATCCAGAGAGGAATGAGTGGCG |
| Keap1 exon 4-5 Rvs | TCAACTGGTCCTGCCCATCGTA |
| Gsta1 Fwd | GTGCCTTGGCAAAAGATAGGACC |
| Gsta1 Rvs | CTTCCAGTAGGTGGATGTCCAC |
